# Strongyloidiasis Beyond the Tropics: Updated Epidemiological Evidence from a Historically Endemic Region in Spain

**DOI:** 10.3390/tropicalmed11030076

**Published:** 2026-03-06

**Authors:** Andrea de Castro-Oliver, Pedro Guevara-Hernández, Javier Guillem, Miquel Moret-Paredes, Alicia Marco-Gabarre, Alicia Lucas-Camps, Celia Prades-Sirvent, David Ruiz-Raga, Ana Ventura Esteve, Maria Amparo Perea Ribis, Marina Llopis Sanchis, Esther Izquierdo García, Hamlet Ghukasyan, María Pallás Cervera, Carmen Visconti Martín, Ana Isabel López Amorós, Angie Gómez Uranga, Sara Vela-Bernal, Ana Isabel de Gracia-Leon, Andreu Belmonte-Domingo, Carolina Pinto-Pla, Ana Ferrer-Ribera, Anaïs Corma-Gómez, María José Galindo, María Rosa Oltra-Sempere, Blanca Belizón, María José Forner, David Navarro, Isabel Corrales, Carlos Bea-Serrano

**Affiliations:** 1 Infectious Disease Unit, Internal Medicine Department, Hospital Clínico Universitario de Valencia, 46010 Valencia, Spain; andreadecasoli@gmail.com (A.d.C.-O.);; 2 INCLIVA Biomedical Research Institute, 46010 Valencia, Spain; 3 Internal Medicine Department, Hospital Francesc de Borja, 46702 Gandía, Spain; pegueher@gmail.com (P.G.-H.);; 4 Microbiology Service, Hospital Clínico Universitario de Valencia, 46010 Valencia, Spain; 5 Internal Medicine Department, Hospital Virgen de los Llirios, 03804 Alcoy, Spain; 6 Pharmacology Department, Faculty of Medicine and Odontology, University of Valencia, 46010 Valencia, Spain

**Keywords:** *Strongyloides stercoralis*, neglected tropical disease, temperate Europe, autochthonous cases, migrant health, screening, immunosuppression

## Abstract

Strongyloidiasis is traditionally regarded as a tropical disease; however, in the Valencian Community (Spain), it has historically been linked to localized endemic foci considered of declining relevance. This was a multicenter regional case series study across three hospitals including patients ≥16 years with strongyloidiasis defined by positive serology and/or parasitological confirmation diagnosed from 2015 to 2024. A total of 301 patients were included (median age 53 years (quartile 1–quartile 3, 40–72); 135 (44.9%) female). Most cases were autochthonous (176/299, 58.9%), while 123/299 (41.1%) occurred in migrants, predominantly from Latin America. Symptoms were present in 165/296 (55.7%), most frequently cutaneous (68/296, 23.0%) and gastrointestinal (62/296, 20.9%). Eosinophilia (>500 cells/µL) was observed in 144/298 (48.3%) and severe infection (hyperinfection syndrome) in 7/294 (2.4%). Annual diagnoses increased over time, with a significant temporal trend by case origin (*p* < 0.001), reflecting an increasing contribution of imported infections, whereas trends by sex (*p* = 0.068) and immune status (*p* = 0.926) were not significant. Stool-based methods demonstrated a low diagnostic yield (microscopy 7/157, 4.5%; culture 21/136, 15.4%; rtPCR 2/27, 7.4%). These findings document the sustained detection of cases classified as autochthonous beyond historically recognized foci and an increasing proportion of imported diagnoses in a temperate European setting.

## 1. Introduction

Strongyloidiasis is caused predominantly by *Strongyloides stercoralis*, a soil-transmitted nematode with worldwide distribution. Despite this broad presence, it remains one of the most neglected tropical diseases [1]. Estimates of the global disease burden vary widely. Frequently cited figures suggest 30–100 million infected individuals [2], although the evidentiary basis for these early estimates has been questioned. More recent spatial modeling studies have proposed substantially higher numbers, exceeding 600 million infections worldwide [3]. These discrepancies largely reflect differences in methodological approaches, including reliance on limited empirical data, modeling assumptions, and variation in diagnostic strategies. Overall, the true global burden remains uncertain and is likely underestimated [3].

Diagnosis of strongyloidiasis is challenging due to intermittent larval excretion and the low sensitivity of conventional stool microscopy [1,4,5,6]. More sensitive methods include the Baermann technique and agar plate culture, while real-time polymerase chain reaction (rtPCR) can achieve higher sensitivity in selected settings, although performance varies [7,8]. Combined diagnostic approaches are therefore recommended. In non-endemic regions, serology remains the principal screening tool, particularly in immunosuppressed individuals, as reflected in current guidelines [9].

*S. stercoralis* has a unique life cycle that includes autoinfection, allowing chronic infection to persist for decades without re-exposure [10]. Most infections are asymptomatic or associated with non-specific symptoms, with eosinophilia often representing the only clue to infection [5]. However, in immunocompromised individuals—including those with hematological malignancies, human T-cell lymphotropic virus type 1 (HTLV-1) infection, organ transplantation, or receiving immunosuppressive therapies, especially corticosteroids—uncontrolled parasite proliferation may lead to hyperinfection syndrome or disseminated disease, conditions associated with high mortality [11]. Consequently, major guidelines recommend screening at-risk individuals for strongyloidiasis prior to initiating immunosuppressive therapy [9,12]. Ivermectin is the treatment of choice and achieves cure rates above 80%, whereas prolonged daily therapy is required in severe disease [9,13,14]. Albendazole is considered a less effective alternative. Recent WHO recommendations also support ivermectin mass drug administration in endemic areas with ≥5% prevalence, alongside sanitation and preventive measures [15].

Although traditionally considered a tropical disease, strongyloidiasis is increasingly identified in temperate regions due to migration, travel, and expanding immunosuppression [16,17]. While zoonotic transmission from dogs has been proposed as a potential contributor to autochthonous cases [18,19], molecular data indicate largely distinct canine and human *Strongyloides* lineages [20], with only limited evidence of cross-transmission [20]. In Spain, both imported and autochthonous infections have been described [16,17]. Prevalence among migrants from endemic areas ranges from 10 to 22% [21]. Autochthonous transmission was historically thought to be rare and largely confined to the *La Safor* region of Valencia, where prevalences of approximately 0.9% in hospital populations and over 12% among rice-field workers were reported [22,23]. While some authors have classified Spain as endemic, others have suggested a geographically restricted focus [16,24]. A systematic review reported that 97% of autochthonous cases occurred in Valencia, mainly among elderly male agricultural workers, with an apparent decline over time [16]. More recent studies, however, have documented locally acquired infections in other regions, including 33 cases from Asturias without travel to endemic areas [25], and reports from southern Alicante confirming the coexistence of imported and autochthonous disease and supporting targeted screening strategies [26]. However, epidemiological data from the Valencian Community remain limited. Comprehensive population-based studies have been confined to *La Safor*, with the most recent dating from 2004 [27], while subsequent evidence derives mainly from small single-center series [14,26]. In a context of increasing immunosuppressive therapies and population mobility, updated regional data are needed to inform screening and management strategies.

This study aimed to describe the temporal trends and demographic characteristics of diagnosed strongyloidiasis cases in the province of Valencia, a non-endemic setting, with particular focus on patterns compatible with autochthonous transmission.

## 2. Materials and Methods

### 2.1. Study Design and Setting

This was a regional case series study across three hospitals in the Valencian Community, Spain: Hospital Clínico Universitario de Valencia (Valencia), Hospital Francesc de Borja (Gandía), and Hospital Virgen de los Lirios (Alcoy). The study period extended from January 2015 to December 2024. We included all patients aged ≥ 16 years with evidence of *S. stercoralis* infection, defined by either a positive serological test or parasitological confirmation.

### 2.2. Data Collection and Laboratory Procedures

Data were obtained through retrospective review of electronic medical records. Collected variables included demographic characteristics (age, sex, country of birth, and postal code of residence), comorbidities, coinfections (HIV, HBV, HCV, HTLV-1, and other parasitic infections), clinical presentation and laboratory findings. Patients were classified as migrants if born in *Strongyloides*-endemic regions and as autochthonous if born in Spain or non-endemic countries and residing in Spain. This classification was based on birthplace and documented exposure history when available and reflects the absence of known exposure to endemic areas rather than confirmed local acquisition. Exposure-related variables (travel to endemic areas, rural residence, or agricultural activity) were recorded when documented.

Clinical presentation at diagnosis was categorized based on documented symptoms: pruritus, abdominal pain, diarrhea, constitutional symptoms (e.g., unexplained weight loss, fatigue), or other symptoms. Laboratory data included eosinophil count and hemoglobin concentration. New immunosuppressive exposures after diagnosis were also recorded.

Serological testing was performed using the Strongyloides IgG/IgM ELISA kit (DRG Diagnostics GmbH, Marburg, Germany) [28]. Optical density (OD) indices were interpreted according to the manufacturer’s instructions: positive (>1.1), indeterminate (0.9–1.1), or negative (<0.9). In routine clinical practice, indeterminate results are repeated when patients return for follow-up evaluation. However, for the purposes of this study, only patients with positive serological results were included, and indeterminate results were not considered diagnostic. Cases with positive serology were classified as infected for study purposes without a prespecified requirement for clinical correlation. Parasitological confirmation was defined as detection of *S. stercoralis* by direct microscopic examination and larval culture of stool samples, as well as molecular detection using a multiplex real-time polymerase chain reaction assay (Allplex^TM^ GI-Helminth Assay, Seegene, Seoul, Republic of Korea) [29], when available. Larval identification in other biological samples, such as tissue biopsies or bronchoalveolar lavage fluid, was also considered confirmatory.

### 2.3. Outcomes and Definitions

The primary outcome was the annual number of diagnosed strongyloidiasis cases recorded in the participating hospitals during the study period. Eosinophilia was defined as >0.5 × 10^9^/L, and anemia as hemoglobin below age- and sex-adjusted laboratory reference values. Immunosuppression at diagnosis was defined by the presence of immunocompromising conditions or therapies, including corticosteroids, biologic agents, other immunosuppressive therapies, chemotherapy, solid organ or hematopoietic stem cell transplantation, hematological malignancy or advanced HIV infection. Hyperinfection syndrome was defined as an accelerated autoinfective cycle with markedly increased parasitological and/or histological detection of larvae (including abundant larvae in stool and/or tissue identification in gastrointestinal biopsies), limited to the gastrointestinal and pulmonary systems and associated with severe gastrointestinal or respiratory symptoms. Disseminated strongyloidiasis was defined as larval involvement of organs beyond the usual life cycle, with systemic complications.

### 2.4. Statistical Analysis

Categorical variables are presented as frequencies and percentages, and continuous variables as mean ± standard deviation (SD) or median [quartile 1–quartile 3 (Q1–Q3)], according to distribution assessed using the Shapiro–Wilk test. Between-group comparisons were conducted using Student’s *t* test or the Mann–Whitney U test for continuous variables, and the chi-square or Fisher’s exact test for categorical variables, as appropriate. For comparisons involving more than two groups, one-way ANOVA or Kruskal–Wallis tests were applied. A two-sided *p* value < 0.05 was considered statistically significant. Temporal changes in the proportion of strongyloidiasis diagnoses by sex, case origin, and immune status were evaluated using the Cochran–Armitage chi-square test for linear trend. Geographical distribution of cases was visualized using Datawrapper software (Datawrapper GmbH, Berlin, Germany), based on patients’ postal code of residence. Statistical analyses were performed using R software (version 4.3.2, R Foundation for Statistical Computing, Vienna, Austria).

## 3. Results

### 3.1. Study Population and Epidemiological Profile

A total of 301 patients were included. Infection was confirmed by positive serology in 293/301 patients (97.3%) and by parasitological detection alone in 8/301 (2.7%). Median [Q1–Q3] age was 53 [40–72] years, and 135 (44.9%) patients were women. Most cases were autochthonous (176/299, 58.9%), while 123/299 (41.1%) were migrants, predominantly from Latin America. Among autochthonous cases, travel to tropical areas was infrequently documented (7/121, 5.8%). Occupational information was available for 68 autochthonous patients, of whom 15 (22.1%) reported agricultural work. Serological screening coverage was high for HIV (79.8%), hepatitis B virus (83.0%), and hepatitis C virus (82.1%), whereas testing for HTLV-1 was performed in only 8 (2.7%) patients, all of whom had negative results. At diagnosis, 53/290 patients (19.3%) were immunocompromised, most frequently due to corticosteroid therapy (7.6%). Baseline characteristics are summarized in Table 1.

### 3.2. Temporal Trends in Strongyloidiasis Cases

**Figure 1:**
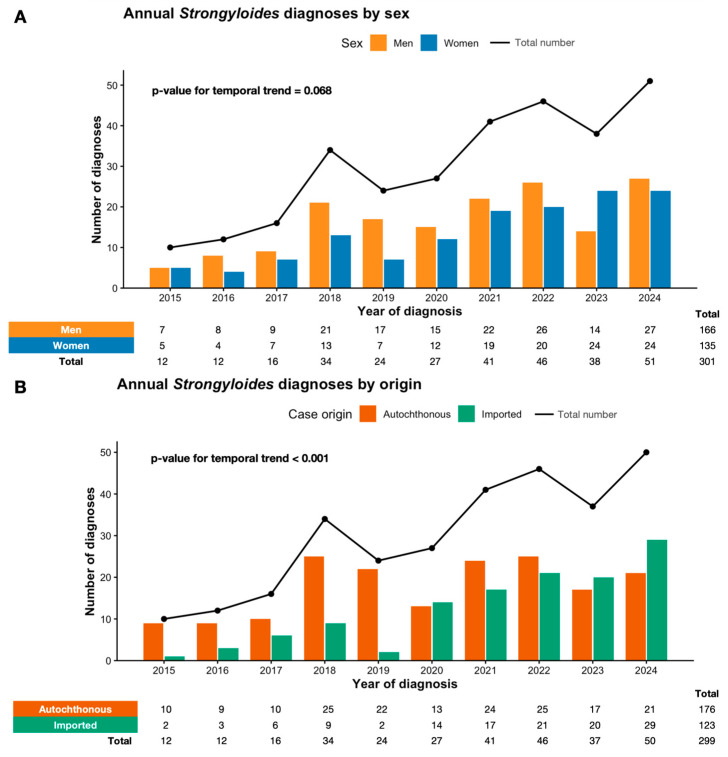

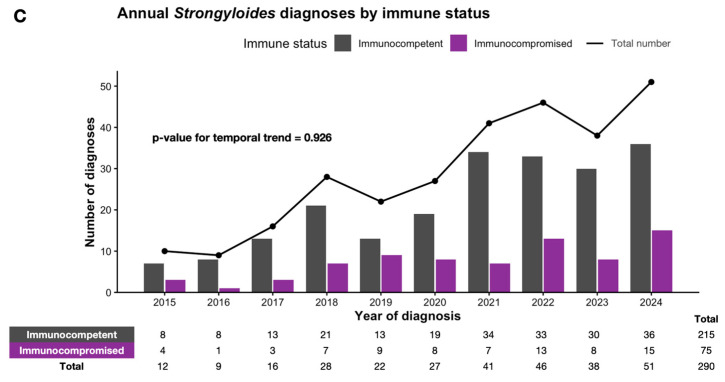
Temporal trends in strongyloidiasis diagnoses between 2015 and 2024. Annual case counts are shown by sex (**A**), case origin (**B**), and immune status (**C**). Bars represent subgroup-specific diagnoses, and the black line indicates total annual diagnoses. Overall, the number of diagnosed cases increased over time. A statistically significant temporal trend was observed according to case origin (*p* < 0.001), reflecting a progressive increase in the number and proportion of imported cases, while cases classified as autochthonous continued to be identified throughout the study period. No significant temporal trends were observed according to sex (*p* = 0.068) or immune status (*p* = 0.926). Temporal trends were assessed using the Cochran–Armitage test for linear trend.

Between 2015 and 2024, the annual number of diagnosed strongyloidiasis cases increased over time, with higher case counts observed from 2018 onwards (Figure 1). Over the same period, the total number of *Strongyloides* serological tests performed per year also increased, from 8 tests in 2015 to 661 in 2024, with a total of 2907 serologies conducted during the study period. When stratified by sex, annual diagnoses rose in both men and women. Although no statistically significant temporal trend was identified in sex distribution (*p* = 0.068), an increase in the absolute number of diagnoses among women was observed during the study period, while men accounted for a greater number of cases in all years (Figure 1A).

A statistically significant temporal trend was identified according to case origin (*p* < 0.001). Autochthonous cases predominated during the initial years of the study, whereas the number and proportion of imported infections increased progressively over time, particularly in the later study period (Figure 1B).

No significant temporal trend was observed according to immune status (*p* = 0.926). Diagnoses among immunocompetent and immunocompromised individuals increased in parallel, with immunocompetent patients representing the majority of cases across all study years (Figure 1C).

### 3.3. Geographical Distribution and Differences Across Study Centers

**Figure 2:**
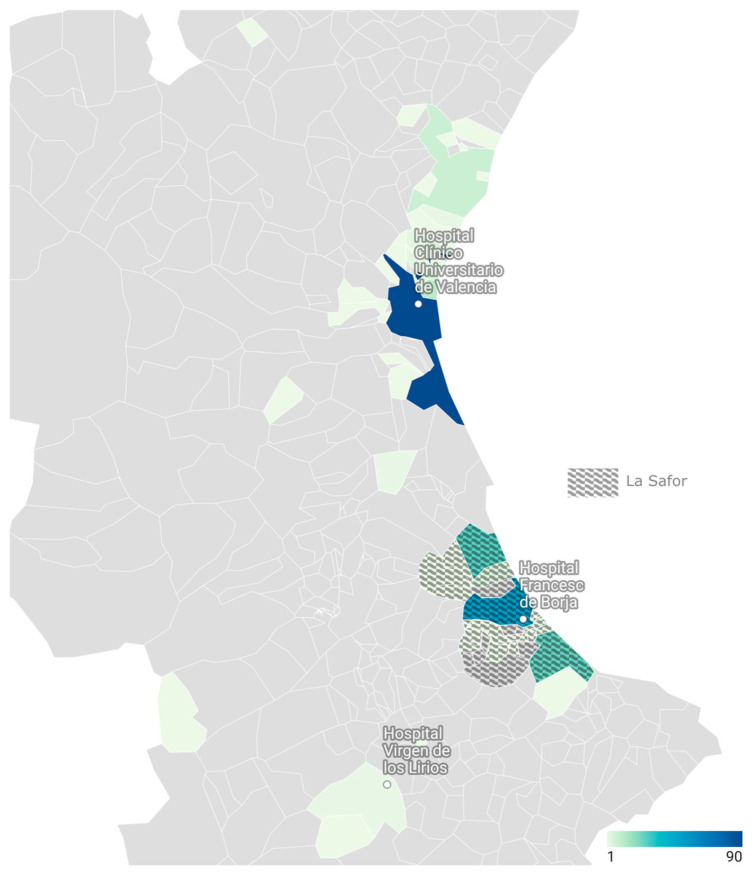
Geographical distribution of strongyloidiasis cases diagnosed in the study centers from 2015 to 2024. The geographical distribution presented reflects the number of diagnosed cases according to postal code of residence and should not be interpreted as a measure of population-based prevalence, as denominator data were not available.

The geographical distribution of strongyloidiasis cases diagnosed during the study period across the three participant centers is shown in Figure 2.

Significant differences in epidemiological profile were observed between centers (Table S1). Hospital Francesc de Borja, located in the historically endemic area of La Safor, included an older population (median age 68 years vs. 45–46 years, *p* < 0.001) and a lower proportion of migrant patients than the other centers (27% vs. 52–75%, *p* < 0.001). Diagnostic testing at Francesc de Borja was mainly prompted by clinical suspicion or isolated eosinophilia (73%), whereas screening-based indications were more frequent at the other centers (42–50%) (*p* < 0.001). The proportion of immunocompromised hosts also differed between centers (34% at Hospital Clínico Universitario de Valencia and 17% at Hospital Francesc de Borja, with no cases at Hospital Virgen de los Lirios; *p* = 0.002).

### 3.4. Clinical Profile and Parasitological Diagnosis

More than half of the patients (165/296, 55.7%) presented symptoms at diagnosis, with cutaneous (68/296, 23.0%) and gastrointestinal (62/296, 20.9%) manifestations as the most frequent clinical presentations. At diagnosis, eosinophilia (>500 cells/µL) was observed in 144/298 patients (48.3%), anemia in 49/298 (16.4%), and both abnormalities in 15/298 patients (5.0%). Seven cases of severe strongyloidiasis were recorded (2.3%), all corresponding to hyperinfection with colonic involvement; no disseminated infections were observed. Detailed clinical characteristics are displayed in Table 2.

Baseline ELISA optical density (OD) index values were available for 192 of the 293 patients with positive serology (65.5%). Nearly one-third of patients (28.1%) with available baseline serological data had weakly positive ELISA results. Direct stool microscopy, larval culture, and stool rtPCR were performed in 157 (52.2%), 136 (45.2%), and 27 (9.0%) cases, respectively. Positive results are presented in Table 2. All patients with parasitological confirmation who underwent serological testing had concomitant positive serology; only eight patients with positive parasitological results did not undergo serological testing. Five patients (2.0%) underwent intestinal biopsy, four of whom showed histological features of colitis; larvae were directly visualized in one specimen. A single cutaneous biopsy revealed a migrating nematode larva compatible with cutaneous *larva migrans* in a patient with concomitant toxocariasis, confirmed by positive *Toxocara* serology.

### 3.5. Comparison Between Migrant and Autochthonous Patients

Baseline characteristics and outcomes according to case origin are summarized in Table 3. Autochthonous patients were significantly older and had a higher comorbidity burden compared with migrant patients. In contrast, coinfection with other parasites was markedly more frequent among migrants. No significant differences were observed between groups regarding sex distribution, immunocompromised status at any time, presence of symptoms at diagnosis, eosinophil counts, baseline ELISA OD index or severe infection.

### 3.6. Comparison According to Immune Status

Baseline characteristics and outcomes according to immune status are summarized in Table 4. Immunocompromised patients had a significantly higher comorbidity burden. At diagnosis, they presented with lower eosinophil counts and lower baseline OD indices compared with immunocompetent individuals. HIV and HCV coinfection was more frequent among immunocompromised patients whereas other parasitic infections were more common in immunocompetent patients. No significant differences were observed regarding age, sex, migration status, symptoms at diagnosis or severe infection.

## 4. Discussion

In this population-based case series, we describe the temporal evolution and clinical–epidemiological characteristics of diagnosed strongyloidiasis cases in the province of Valencia over a 10-year period. The annual number of diagnoses increased substantially over time, particularly in the most recent years, a trend largely driven by a progressive rise in imported cases, while the number of autochthonous cases remained relatively stable throughout the study period. With regard to sex distribution, no clear linear temporal trend was observed, although a higher proportion of female cases was noted in the later years of the study period. The distribution by immune status remained stable over time. Overall, clinical and laboratory characteristics at diagnosis were largely comparable across groups, with differences mainly confined to age, comorbidity burden, and specific coinfection patterns according to migration and immune status. Together, these findings provide an updated overview of the contemporary epidemiology of strongyloidiasis diagnoses in this non-endemic setting.

### 4.1. Autochthonous Cases: Re-Examining the “Non-Endemic” Paradigm

In our cohort, more than half of the patients were Spain-born and reported no history of travel to endemic regions, especially those diagnosed at Hospital Francesc de Borja (Gandía) in the historically endemic *La Safor* area, but also in adjacent health departments, including Valencia city (Hospital Clínico Universitario de Valencia) and Alcoy (Hospital Virgen de los Lirios). Notably, the median age of autochthonous patients in our cohort remained high, consistent with historical series, supporting the hypothesis of long-standing infections maintained through autoinfection [30]. However, only a small minority of patients reported agricultural work, and documented travel to tropical areas was uncommon. In contrast to other series [16,26], male predominance among autochthonous cases was not significant in our series. Although the previously mentioned systematic review of endemic cases in Spain [16] showed a decrease in the number of cases reported since 2011, in our series most cases were diagnosed in recent years (2015–2024), with a progressive increase in identified cases over time. These findings align with more recent emerging evidence from other Spanish regions. Rodríguez-Pérez et al. recently reported the first substantial series of autochthonous strongyloidiasis in Asturias, identifying 33 locally acquired cases between 2016 and 2024 in an area with no previously recognized endemicity [25]. Interestingly, affected individuals were younger, not predominantly male, and infections were linked mainly to gardening rather than professional agricultural activity. This underscores the limitations of relying on classical exposure profiles and suggests that infection may occur through less well-defined environmental exposures or recreational contact with contaminated soil, and directly questions recent literature suggesting that Spain is essentially non-endemic for strongyloidiasis [24], with autochthonous cases being merely anecdotal and limited to a highly localized rice-farming focus in *La Safor* [16].

### 4.2. Temporal Trends: Rising Imported Cases Amid Stable Autochthonous Cases

Temporal trends in our study show a statistically significant shift in case origin over the past decade. Autochthonous cases predominated in the initial years (2015–2019), whereas the number and proportion of imported infections increased progressively over time, particularly in the later study period (*p* < 0.001). By the end of the study period, migrant patients (primarily from Latin American endemic regions) represented an increasingly large share of annual diagnoses in our hospitals, potentially influenced by screening practices and healthcare access. While this pattern may reflect ongoing migration from endemic areas [17], it should be interpreted cautiously. Absolute case numbers remained modest, and changes in screening intensity, increased awareness, or modifications in hospital testing practices—particularly among migrant and immunosuppressed populations—may have contributed to the observed temporal variation. Importantly, the rise in imported cases did not coincide with a decline in cases classified as autochthonous, which continued to be identified throughout the study period.

Although men accounted for the majority of cases throughout the study period, temporal analysis showed an increase in diagnoses among women over time. While this trend did not reach statistical significance, the progressive rise in female cases observed in recent years [31] may similarly reflect evolving screening practices and healthcare access rather than true differences in exposure.

No significant temporal trend was observed according to immune status. As previously described in other series [32], immunocompetent individuals represented the majority of diagnosed cases across all study years, and the proportion of immunocompromised patients remained relatively stable over time. This pattern may reflect stable screening practices in high-risk clinical settings [31].

### 4.3. Clinical Profile: Autochthonous vs. Imported Cases

Despite clear epidemiological differences, with autochthonous patients being older and presenting a higher comorbidity burden, the clinical presentation of autochthonous and migrant patients was largely comparable. Both groups exhibited similar symptom patterns, laboratory abnormalities, and severe disease presentation. These findings are consistent with prior Spanish studies reporting equivalent clinical behavior of locally acquired and imported strongyloidiasis [26]. Hyperinfection remained rare overall (with no cases of disseminated strongyloidiasis) and occurred mainly in individuals with substantial comorbidities or corticosteroid exposure, reinforcing the central role of host immune status rather than geographic origin in determining prognosis. The immunocompromised group, however, was heterogeneous and included patients receiving corticosteroids, those with malignancies, transplant recipients, and individuals with advanced HIV infection. The relatively small number of cases within each specific subgroup precluded further stratified analyses to explore differential risks according to type of immunosuppression.

### 4.4. Co-Infections: High T. cruzi and Blastocystis, Low HTLV-1 Testing

Coinfection with *T. cruzi* was frequent in our cohort, particularly among patients originating from Bolivia. This association is well established and reflects overlapping epidemiology rather than biological interaction. A Spanish case–control study demonstrated a significant association between Chagas disease and *S. stercoralis* seropositivity even after adjustment for confounders, supporting systematic reciprocal screening [33]. In routine practice, *Strongyloides* serology is often automatically performed following a Chagas diagnosis, which likely explains both the high prevalence of coinfection and the predominance of Bolivian patients in our series. *Blastocystis hominis* was also frequently detected, as reported in other cohorts [34], possibly reflecting shared fecal–oral exposure pathways and underlying polyparasitism. In contrast, HTLV-1 testing was performed in only a small proportion of patients, and no cases were identified. Given the well-established association between HTLV-1 coinfection and severe strongyloidiasis, including hyperinfection, the limited testing in our cohort precludes meaningful assessment of its contribution to disease severity. This represents an important limitation and highlights the need for greater awareness and systematic consideration of HTLV-1 screening in patients with strongyloidiasis [35].

### 4.5. Implications for Screening

In the context of these findings, our results are consistent with current expert consensus recommendations for the management of strongyloidiasis in intermediate- to low-risk settings such as the province of Valencia [9]. The sustained identification of autochthonous cases and the occurrence of infection among immunocompromised patients highlight the continued relevance of maintaining a high index of suspicion in these populations, particularly prior to the initiation of immunosuppressive therapies. In line with existing guidelines, systematic screening in immunocompromised individuals may therefore remain a reasonable preventive approach in this setting. Conversely, in immunocompetent individuals without planned immunosuppression, our data do not support the need for universal screening, and diagnostic testing may be more appropriately guided by clinical or laboratory findings such as eosinophilia, in accordance with expert recommendations [9].

### 4.6. Diagnostic Considerations

In our cohort, the vast majority of diagnoses were based on serology, reflecting routine clinical practice in non-endemic European settings where ELISA constitutes the primary screening tool [9]. Serological assays for *S. stercoralis* show variable performance, with reported sensitivity ranging from 60 to 90% and specificity between 80 and 98% [36,37], and cross-reactivity with other helminths has been described [28]. As systematic parasitological confirmation was not performed in all cases, and nearly one-third of cases showed weakly positive optical density values, some degree of overestimation of true infection cannot be excluded.

### 4.7. One Health Perspectives and Emerging Diagnostic Strategies

A broader One Health perspective may contribute to improved strongyloidiasis control, particularly in temperate settings where environmental, socio-economic, and migratory factors intersect [15]. Although zoonotic transmission from dogs has been proposed [18,19,38], recent molecular data suggest genetic separation between most canine and human *Strongyloides* populations, with limited evidence of sustained cross-transmission [20]. Nonetheless, integrated surveillance involving human health services, veterinary assessment when appropriate, and environmental risk evaluation may enhance understanding of local transmission patterns [15]. In parallel, novel diagnostic strategies such as nanobiosensor-based platforms have been proposed to improve the detection of parasitic infections, including *S. stercoralis*, particularly in low-intensity or hard-to-diagnose cases. These technologies aim to enhance sensitivity through nanoparticle-based signal amplification and portable point-of-care systems, potentially integrated with digital health tools and artificial intelligence-assisted analysis. Although promising, their implementation faces important challenges, including assay standardization, multicenter validation, regulatory approval, and cost-effectiveness. Their added value in low-prevalence temperate settings remains to be demonstrated [39].

### 4.8. Strengths and Limitations

This study has limitations inherent to its retrospective design. The use of routinely collected clinical data resulted in incomplete availability of some variables, potentially limiting a more detailed characterization of individual cases. In particular, alcohol use disorder, a recognized risk factor for severe strongyloidiasis, was not systematically documented and could therefore not be analyzed. Classification of cases as autochthonous or imported was based on birthplace and documented exposure history, and some degree of misclassification cannot be excluded. Long-standing chronic infections acquired decades earlier remain a plausible explanation, particularly among elderly patients classified as autochthonous, precluding confirmation of recent local transmission. Moreover, as this study is based on diagnosed cases, the observed temporal trends reflect changes in case detection and clinical practice rather than direct estimates of incidence or transmission dynamics. The study design does not allow inference regarding ongoing transmission or true changes in disease burden. Finally, diagnostic workup was not fully standardized across centers, reflecting real-world clinical practice and introducing heterogeneity in parasitological and serological assessment.

Despite these limitations, this study has several important strengths. It represents one of the largest contemporary case series of strongyloidiasis reported from a non-tropical European setting and spans a decade of diagnoses across multiple hospitals. The long observation period and inclusion of both autochthonous and migrant patients enabled a robust description of temporal trends and comparative epidemiological patterns. Importantly, the retrospective, real-world nature of the study population allowed capture of routine clinical diagnoses over time, providing a comprehensive overview of diagnosed strongyloidiasis in a non-endemic setting.

## 5. Conclusions

This multicenter case series provides updated regional data on 301 diagnosed cases of strongyloidiasis over a 10-year period in a temperate European region. We observed sustained detection of cases classified as autochthonous beyond historically recognized foci, alongside a statistically significant increase in the proportion of imported diagnoses over time. Clinical presentation was comparable between autochthonous and migrant patients, and hyperinfection remained uncommon. Serology constituted the main diagnostic tool, while stool-based methods showed limited diagnostic yield in routine practice. These findings support the continuation of targeted screening strategies in immunocompromised individuals and migrant populations in temperate settings. Future prospective studies incorporating standardized diagnostic algorithms and population-based seroprevalence studies are warranted to more accurately define the true burden of infection and clarify transmission dynamics in this region.

## Figures and Tables

**Table 1:** Main characteristics of 301 patients with strongyloidiasis diagnosed in the study centers from 2015 to 2024.

		n * (%) or Median [IQR]
Female sex		135/301 (44.9%)
Age (years)		53.0 [40.0–72.0]
Migrant		123/299 (41.1%)
Country of origin	Bolivia	44/123 (35.8%)
	Ecuador	14/123 (11.4%)
	Colombia	11/123 (8.9%)
	Honduras	6/123 (4.9%)
	Equatorial Guinea	5/123 (4.1%)
	Other countries	43/123 (35.0%)
Autochthonous		176/299 (58.9%)
Agricultural worker		15/68 (22.1%)
Travel to tropical area		7/121 (5.8%)
***Country of destination***	Colombia	2/7 (33.0%)
	Equatorial Guinea	1/7 (16.7%)
	India	1/7 (16.7%)
	Mexico	1/7 (16.7%)
	Nicaragua	1/7 (16.7%)
	Dominican Republic	1/7 (16.7%)
Unknown exposure risk		154/176 (87.5%)
Residential setting	Urban	160/263 (60.8%)
	Rural	20/263 (7.6%)
	Mixed	83/263 (31.6%)
Comorbidities	Solid neoplasia	17/290 (5.9%)
	Lymphoma/leukemia	5/290 (1.7%)
	Diabetes mellitus	40/290 (13.8%)
	Cardiovascular disease ^1^	38/290 (13.1%)
	Chronic kidney disease ^2^	53/290 (18.3%)
	Dementia	8/290 (2.8%)
	COPD/Asthma	25/290 (8.6%)
	Liver disease	9/290 (3.1%)
	Connective tissue disease	19/290 (6.6%)
	Charlson comorbidity index	2 [0–4]
Coinfections	HIV ^3^	11/240 (4.6%)
	HBV (resolved infection)	19/250 (7.6%)
	HCV	5/247 (2.0%)
	*Trypanosoma cruzi*	22/301 (7.3%)
	*Blastocystis hominis*	22/301 (7.3%)
	Other parasitic infections	8/301 (2.7%)
Baseline immunocompromised status ^4^		53/290 (19.3%)
	Corticosteroids	22/290 (7.6%)
	Biologic therapy	8/290 (2.8%)
	Other immunosuppressants	6/290 (2.1%)
	Solid organ transplantation	3/290 (1.0%)
	Chemotherapy	7/290 (2.3%)
	Hematological malignancy	5/290 (1.7%)
	HSCT	1/290 (0.3%)
	AIDS	6/290 (2.1%)
	Other immunosuppression	2/290 (0.7%)

* Values are reported as n/N (%), with denominators corresponding to available data for each variable; denominators may vary due to missing information. Serological data from Hospital Francesc de Borja were unavailable for the period between May 2015 and October 2016. ^1^ Cardiovascular disease includes a history of myocardial infarction, congestive heart failure, peripheral vascular disease and stroke/transient ischemic attack. ^2^ Moderate to severe chronic kidney disease: dialysis, status post kidney transplant, creatinine > 3 mg/dL. ^3^ Median CD4 count 445 cells/mL [270–572]. ^4^ Presence of immunosuppression at diagnosis. AIDS, acquired immunodeficiency syndrome; COPD, chronic obstructive pulmonary disease; HBV, hepatitis B virus; HCV, hepatitis C virus; HIV, human immunodeficiency virus; HSCT, hematopoietic stem cell transplantation.

**Table 2:** Baseline clinical characteristics and microbiological test results of 301 patients with strongyloidiasis diagnosed in the study centers from 2015 to 2024.

Clinical Characteristics		n * (%) or Median [IQR]
Reason for test request	Clinical suspicion	99/273 (36.3%)
	Isolated eosinophilia	85/273 (31.1%)
	Screening ^1^	89/273 (32.6%)
Symptoms at diagnosis	Any symptoms	165/296 (55.7%)
	Pruritus	61/296 (20.6%)
	Exanthema	29/296 (9.8%)
	Abdominal pain	48/296 (16.2%)
	Diarrhea	40/296 (13.5%)
	Other gastrointestinal symptoms	19/296 (6.4%)
	Weight loss	23/296 (7.8%)
	Respiratory symptoms	12/296 (4.1%)
	Other symptoms	9/296 (3.0%)
Baseline laboratory values	Eosinophils count (cells/µL)	430.0 [160.0–1150.0]
	Hemoglobin (g/dL)	13.2 [12.3–14.6]
Severe infection ^2^		7/294 (2.4%)
Baseline diagnostic test results		
ELISA OD index	1.1–1.5	54/192 (28.1%)
	>1.5–3	68/192 (35.4%)
	>3	70/192 (36.3%)
Direct stool microscopy	Positive	7/157 (4.5%)
Larval culture	Positive	21/136 (15.4%)
Stool rtPCR	Positive	2/27 (7.4%)

* Values are reported as n/N (%), with denominators corresponding to patients in whom each test was performed and results were available. For the ELISA optical density index, the denominator corresponds to patients with positive serology in whom OD data were available. For direct stool microscopy, larval culture, and stool rtPCR, denominators represent the number of patients tested with available results. ^1^ Screening included migrants from endemic regions, evaluation before or during pharmacological- or malignancy-related immunosuppression, and organ donation. ^2^ Severe infection refers to *Strongyloides* hyperinfection syndrome; no cases of disseminated strongyloidiasis were observed. OD, optical density; rtPCR, polymerase chain reaction.

**Table 3:** Main characteristics of strongyloidiasis in autochthonous versus migrant populations.

		Autochthonous n = 176 *	Migrant n = 123 *	*p*-Value
Female sex		71/176 (40.3%)	63/123 (51.2%)	0.063
Age (years)		67.5 [47.0–78.5]	43.00 [37.0–53.0]	<0.001
Charlson comorbidity index		3.0 [1.5–5.0]	0.0 [0–2.0]	<0.001
Coinfections	HIV	3/133 (2.3%)	8/106 (7.5%)	0.065
	HBV (resolved infection)	7/141 (5.0%)	12/108 (11.1%)	0.070
	HCV	3/142 (2.1%)	2/104 (1.9%)	1.000
	Other parasitic infection	14/176 (8.0%)	37/123 (30.1%)	<0.001
Immunocompromised at any time		46/167 (27.5%)	29/121 (24.0%)	0.495
Symptoms at diagnosis		78/171 (45.6%)	52/123 (42.3%)	0.570
Eosinophils count at diagnosis (cells/µL)		520.0 [160.0–1300.0]	310.0 [150.0–1000.0]	0.088
Hemoglobin at diagnosis (g/dL)		13.2 [11.9–14.6]	13.3 [12.5–14.8]	0.2
Baseline ELISA OD index		2.23 [1.40–5.12]	2.47 [1.52–7.60]	0.2
Positive larval culture		15/87 (17.2%)	7/50 (14.0%)	0.619
Severe infection ^1^		4/173 (2.3%)	3/121 (2.4%)	0.98

* Values are reported as n/N (%), with denominators corresponding to available data for each variable; denominators may vary due to missing information. ^1^ Severe infection refers to *Strongyloides* hyperinfection syndrome; no cases of disseminated strongyloidiasis were observed.

**Table 4:** Main characteristics of strongyloidiasis according to immune status.

		Immunocompetent n = 215 *	Immunocompromised n = 75 *	*p*-Value
Female sex		108/215 (50.2%)	24/75 (32.0%)	0.006
Age (years)		52.0 [40.0–74.0]	53.0 [41.0–68.0]	0.9
Migrant		92/213 (43.2%)	29/75 (38.7%)	0.495
Charlson comorbidity index		1.0 [0.0–4.0]	3.0 [1.0–4.0]	0.003
Coinfections	HIV	0/166 (0%)	11/69 (15.9%)	0.001
	HBV (resolved infection)	14/175 (8.0%)	5/70 (7.1%)	0.821
	HCV	0/173 (0.0%)	5/69 (7.2%)	0.002
	Other parasitic infection	44/176 (25.0%)	6/62 (9.7%)	0.011
Symptoms at diagnosis		104/212 (49.1%)	27/74 (36.5%)	0.062
Eosinophils count at diagnosis (cells/µL)		520.0 [180.0–1200.0]	245.0 [110.0–780.0]	0.030
Hemoglobin at diagnosis (g/dL)		13.20 [12.4–14.6]	13.2 [12.0–14.6]	0.5
Baseline ELISA OD index		2.5 [1.5–6.8]	1.7 [1.4–3.5]	0.010
Positive larval culture		18/102 (17.6%)	3/31 (9.7%)	0.402
Severe infection ^1^		5/215 (2.3%)	2/75 (2.7%)	1.000

* Values are reported as n/N (%), with denominators corresponding to available data for each variable; denominators may vary due to missing information. ^1^ Severe infection refers to *Strongyloides* hyperinfection syndrome; no cases of disseminated strongyloidiasis were observed.

## Data Availability

The data presented in this study are available from the corresponding author upon reasonable request. Restrictions apply due to patient privacy and ethical considerations.

## Supplementary Materials

The following supporting information can be downloaded at: https://www.mdpi.com/article/10.3390/tropicalmed11030076/s1, Table S1: Differences in epidemiological and reason for referral among the different study centers.

## Abbreviations

The following abbreviations are used in this manuscript:

AIDS	Acquired immunodeficiency syndrome
COPD	Chronic obstructive pulmonary disease
ELISA	Enzyme-linked immunosorbent assay
HBV	Hepatitis B virus
HCV	Hepatitis C virus
HIV	Human immunodeficiency virus
HSCT	Hematopoietic stem cell transplantation
HTLV-1	Human T-cell lymphotropic virus type 1
OD	Optical density
rtPCR	Real-time polymerase chain reaction
