# Supplementary material for: Strongyloidiasis Beyond the Tropics: Updated Epidemiological Evidence from a Historically Endemic Region in Spain
Source: Trop Med Infect Dis. 2026 Mar 6;11(3):76. doi: 10.3390/tropicalmed11030076 (PMC13030033; doi:10.3390/tropicalmed11030076)
Supplement: Supplementary file 1 [file tropicalmed-11-00076-s001.zip › tropicalmed-4147248-supplementary.pdf]

Supplementary material

Table S1. Differences in epidemiological and reason for referral among the different study centers.

		Study center			
		**Hospital Clínico de Valencia n=160***	**Hospital Francesc de Borja n=137***	**Hospital Virgen de los Lirios n=4***	**p-value**
**Female sex**		79/160 (49.4%)	55/137 (40.1%)	1/4 (25.0%)	0.2
**Age (years)**		46 [38–59]	68 [50–80]	45 [38–55]	<0.001
**Migrant**		83/159 (52.2%)	37/136 (27.2%)	3/4 (75%)	<0.001
**Immunocompromised at any time**		54/160 (33.8%)	21/126 (16.7%)	0 (0%)	0.002
**Reason for test request**	Clinical suspicion	67/150 (44.7%)	31/119 (26.1%)	1/4 (25%)	<0.001
	Isolated eosinophilia	16/150 (10.7%)	68/119 (57.1%)	1/4 (25%)	
	Screening[1]	67/150 (44.7%)	20/119 (16.8%)	2/4 (50%)	

*Values are reported as n/N (%), with denominators corresponding to available data for each variable; denominators may vary due to missing information. [1] Screening included migrant from endemic regions screening from endemic regions, evaluation before or during pharmacological or malignancy-related immunosuppression, and organ donation.
